# Whole-genome analyses of extended-spectrum or AmpC β-lactamase-producing *Escherichia coli* isolates from companion dogs in Japan

**DOI:** 10.1371/journal.pone.0246482

**Published:** 2021-02-05

**Authors:** Mayo Yasugi, Shingo Hatoya, Daisuke Motooka, Yuki Matsumoto, Shunsuke Shimamura, Hiroyuki Tani, Masaru Furuya, Keiichiro Mie, Masami Miyake, Shota Nakamura, Terumasa Shimada

**Affiliations:** 1 Graduate School of Life and Environmental Sciences, Osaka Prefecture University, Izumisano, Osaka, Japan; 2 Asian Health Science Research Institiute, Osaka Prefecture University, Izumisano, Osaka, Japan; 3 Research Institute for Microbial Diseases, Osaka University, Suita, Osaka, Japan; Zhejiang University, CHINA

## Abstract

The emergence and global spread of extended-spectrum or AmpC β-lactamase (ESBL/AmpC)-producing Enterobacteriaceae in companion animals have led to the hypothesis that companion animals might be reservoirs for cross-species transmission because of their close contact with humans. However, current knowledge in this field is limited; therefore, the role of companion animals in cross-species transmission remains to be elucidated. Herein, we studied ESBL/AmpC-producing Enterobacteriaceae, *Escherichia coli* in particular, isolated from extraintestinal sites and feces of companion dogs. Whole-genome sequencing analysis revealed that (i) extraintestinal *E*. *coli* isolates were most closely related to those isolated from feces from the same dog, (ii) chromosomal sequences in the ST131/C1-M27 clade isolated from companion dogs were highly similar to those in the ST131/C1-M27 clade of human origin, (iii) certain plasmids, such as IncFII/pMLST F1:A2:B20/*bla*_CTX-M-27_, IncI1/pMLST16/*bla*_CTX-M-15_, or IncI1/*bla*_CMY-2_ from dog-derived *E*. *coli* isolates, shared high homology with those from several human-derived Enterobacteriaceae, (iv) chromosomal *bla*_CTX-M-14_ was identified in the ST38 isolate from a companion dog, and (v) eight out of 14 tested ESBL/AmpC-producing *E*. *coli* isolates (i.e., ST131, ST68, ST405, and ST998) belonged to the human extraintestinal pathogenic *E*. *coli* (ExPEC) group. All of the *bla*-coding plasmids that were sequenced genome-wide were capable of horizontal transfer. These results suggest that companion dogs can spread ESBL/AmpC-producing ExPEC via their feces. Furthermore, at least some ESBL/AmpC-producing ExPECs and *bla*-coding plasmids can be transmitted between humans and companion dogs. Thus, companion dogs can act as an important reservoir for ESBL/AmpC-producing *E*. *coli* in the community.

## Introduction

The World Health Organization published a report in 2014 showing very high rates of resistance in bacteria that cause common health-care associated and community-acquired infections worldwide [1]. In 2016, it was estimated that unless action was taken, the number of deaths resulting from antimicrobial resistance (AMR) could balloon to ten million per year by 2050 [2]. AMR bacteria are now one of the most serious issues worldwide. Of these, third-generation cephalosporin-resistant (3GCR) Enterobacteriaceae are of great concern because of an increasing trend in their prevalence globally [3]. 3GCR Enterobacteriaceae produce extended-spectrum or AmpC β-lactamases (ESBL/AmpC) that hydrolyze the β-lactam ring of third-generation cephalosporins [4]. ESBL-producing Enterobacteriaceae emerged during the 1980s. Most of the early ESBLs described were of the TEM or SHV type, mainly found in *Klebsiella pneumoniae*, and were usually associated with nosocomial outbreaks [5]. Since the late 1990s, the incidence of ESBLs of the CTX-M type, particularly in *Escherichia coli*, has increased dramatically, and not only in nosocomial environments, but also in community settings [6,7]. The CTX-M type is currently the most prevalent ESBL worldwide. AmpC genes are located on the chromosome in several Enterobacteriaceae, such as *Enterobacter* spp. and *Citrobacter* spp [8]. Chromosomal AmpC genes were mobilized onto plasmids by insertion sequences, and plasmid-borne AmpC emerged in *K*. *pneumoniae* in the late 1980s [9]. Transmissible plasmids encoding AmpC gene have spread in bacteria that lack or poorly express chromosomal AmpC genes, such as *E*. *coli*, *K*. *pneumoniae*, and *Proteus mirabilis* [8,9]; therefore, plasmid-borne AmpC-producing Enterobacteriaceae are now highly prevalent in humans.

Global concerns have also been raised about the dissemination of ESBL/AmpC-producing Enterobacteriaceae in animals. In livestock animals, poultry and poultry products are considered reservoirs of ESBL/AmpC-producing *Salmonella enterica* and *E*. *coli* because of the high incidence of CMY-2-producing *E*. *coli* in poultry [10]. Many studies suggested that ESBL-producing *E*. *coli* are clonally transferred from livestock to humans, based on typing methods [11–13]. More recently, whole-genome analysis of ESBL/AmpC-producing *E*. *coli* isolated from humans, livestock animals, and meat products suggested that clonal transmission of the *bla* gene is a rare event, while the horizontal transfer of temporally stable *bla*-carrying plasmids is more likely the dominant form of cross-species transmission [11,14]. ESBL/AmpC-producing Enterobacteriaceae are also detected worldwide in companion animals [15–17]. Surveillance in Japan showed that approximately 40% of tested diseased dogs and cats carried 3GCR *E*. *coli* [18,19]. The emergence and spread of ESBL/AmpC-producing Enterobacteriaceae among companion animals pose a risk of the animals being reservoirs for cross-species transmission, because of their close contact with humans [20,21]. Clonal transfer of ESBL-producing Enterobacteriaceae between companion animals and humans in the same household was previously suggested by typing methods [17,22], and more recently by whole-genome analysis [23–25]. However, current knowledge of cross-species transmission is still limited; thus, the precise role of companion animals in cross-species transmission of ESBL/AmpC-producing Enterobacteriaceae is not fully understood. To shed light on this issue, here we studied ESBL/AmpC-producing Enterobacteriaceae, in particular *E*. *coli*, isolated from companion dogs and compared the chromosomal and plasmid sequences with those from humans at the genome-wide scale.

## Materials and methods

### Ethics

Animal samples were collected using protocols approved by the Institutional Review Boards of the Veterinary Medical Center, Osaka Prefecture University. Written informed consent was obtained from the owners. All samples were anonymized before use.

### Bacterial isolates

All clinical specimens characterized in this study were collected at the Veterinary Medical Center, Osaka Prefecture University in 2018. All extraintestinal bacterial isolates were isolated, identified, and tested for antimicrobial susceptibility as part of a clinical diagnosis by Japan Clinical Laboratories (Kyoto, Japan). The isolates that were suspected to be ESBL/AmpC-producing Enterobacteriaceae were provided by Japan Clinical Laboratories, cultured in brain heart infusion (BHI) broth at 37°C overnight, and stored at −80°C with glycerol (final concentration, 20%) for future use. Fecal samples were obtained from 12 dogs. The feces were streaked on MacConkey agar containing 4 μg/ml cefotaxime, and cultured at 37°C overnight. After colonies were subcloned, five colonies underwent further biochemical characterization for species identification. Finally, the identified Enterobacteriaceae isolates were cultured in BHI broth supplemented with 4 μg/ml cefotaxime, and stored at −80°C with glycerol for future use.

### Antimicrobial susceptibility testing

Minimal inhibitory concentration (MIC) assays for ampicillin, cefazolin, cefmetazole, cefotaxime, ceftazidime, ceftriaxone, cefpirome, imipenem, meropenem, levofloxacin, and ciprofloxacin were performed on the isolates according to the broth microdilution method of the Clinical and Laboratory Standards Institute (CLSI) by following instructions in CLSI document M07-A10 [26]. The results were interpreted using CLSI M100-S26 guidelines [27]. The 3GCR isolates underwent further phenotypic testing for the production of ESBL, AmpC, and/or metallo-β-lactamase as follows: ESBL production was confirmed by a disc diffusion assay using a mixture of antibiotics (cefotaxime or ceftazidime) and clavulanic acid; AmpC production was confirmed by a disc diffusion assay using a mixture of antibiotics (cefmetazole, cefotaxime, or ceftazidime) and aminophenylboronic acid; metallo-β-lactamase production was confirmed by a double-disc synergy assay using antibiotics (imipenem or ceftazidime) and sodium mercaptoacetate. The results were interpreted as described previously [27–29].

Genotypes of the ESBL/AmpC-producing Enterobacteriaceae were determined by a direct sequencing method. The isolates were cultured in tryptic soy broth (TSB) supplemented with 4 μg/ml cefotaxime at 37°C overnight. After centrifugation, culture pellets were suspended in distilled water and boiled for 10 min. After another centrifugation step, supernatants were subjected to PCR using GoTaq polymerase (Promega, Madison, WI) with genogroup-specific primers (Table 1). After genogroups were identified, the same samples were subjected to PCR using PrimeSTAR GXL DNA polymerase (Takara, Shiga, Japan) with the genotype-specific primers described in Table 1. After electrophoresis, amplified DNA was extracted with the QIAquick Gel Extraction kit (Qiagen, Hilden, Germany) and sequenced (Macrogen, Kyoto, Japan). Blastp (https://blast.ncbi.nlm.nih.gov/Blast.cgi) was used to find sequence similarities, and to determine the CTX-M and CMY-2 genotype. Single-nucleotide polymorphisms (SNPs) were used to determine the TEM genotype [30,31].

**Table 1:** Primers used in this study.

Primer ID	Sequence	Target	Size (bp)	Ref.
Pr371	5′-CCGTGTCGCCCTTATTCC-3′	TEM genogroup-F	824	[32]
Pr372	5′-AGGCACCTATCTCAGCGA-3′	TEM genogroup-R		
Pr373	5′-ATTTGTCGCTTCTTTACTCGC-3′	SHV genogroup-F	1051	[32]
Pr374	5′-TTTATGGCGTTACCTTTGACC-3′	SHV genogroup-R		
Pr375	5′-GCTGTTGTTAGGAAGTGTGC-3′	CTX-M-1 genogroup-F	516	[33]
Pr376	5′-CCATTGCCCGAGGTGAAG-3′	CTX-M-1 genogroup-R		
Pr377	5′-ACGCTACCCCTGCTATTT-3′	CTX-M-2 genogroup-F	780	[33]
Pr378	5′-GCTTTCCGCCTTCTGCTC-3′	CTX-M-2 genogroup-R		
Pr379	5′-GCAGATAATACGCAGGTG-3′	CTX-M-9 genogroup-F	393	[33]
Pr380	5′-CGGCGTGGTGGTGTCTCT-3′	CTX-M-9 genogroup-R		
Pr393	5′-GCTGCTCAAGGAGCACAGGAT-3′	MOX genogroup-F	520	[34]
Pr394	5′-CACATTGACATAGGTGTGGTGC-3′	MOX genogroup-R		
Pr395	5′-TGGCCAGAACTGACAGGCAAA-3′	CIT genogroup-F	462	[34]
Pr396	5′-TTTCTCCTGAACGTGGCTGGC-3′	CIT genogroup-R		
Pr397	5′-AACTTTCACAGGTGTGCTGGGT-3′	DHA genogroup-F	405	[34]
Pr398	5′-CCGTACGCATACTGGCTTTGC-3′	DHA genogroup-R		
Pr399	5′-AACAGCCTCAGCAGCCGGTTA-3′	ACC genogroup-F	346	[34]
Pr400	5′-TTCGCCGCAATCATCCCTAGC-3′	ACC genogroup-R		
Pr401	5′-TCGGTAAAGCCGATGTTGCGG-3′	EBC genogroup-F	302	[34]
Pr402	5′-CTTCCACTGCGGCTGCCAGTT-3′	EBC genogroup-R		
Pr403	5′-AACATGGGGTATCAGGGAGATG-3′	FOX genogroup-F	190	[34]
Pr404	5′-CAAAGCGCGTAACCGGATTGG-3′	FOX genogroup-R		
Pr513	5′-GACTATTCATGTTGTTGTTATTTC-3′	CTX-M-1 genotype-F	923	[35]
Pr514	5′-TTACAAACCGTTGGTGACG-3′	CTX-M-1 genotype-R		
Pr515	5′-ATGGTGACAAAGAGAGTGCA-3′	CTX-M-9 genotype-F	900	[36]
Pr516	5′-CCCTTCGGCGATGATTCTC-3′	CTX-M-9 genotype-R		
Pr569	5′-ATGATGAAAAAATCGTTATGCT-3′	CMY-2 genotype-F	562	[37]
Pr570	5′-TTATTGCAGCTTTTCAAGAATGCG-3′	CMY-2 genotype-R		
Pr573	5′-ATTCTTGAAGACGAAAGGGC-3′	TEM genotype-F	1091	[38]
Pr574	5′-ATGAGTAAACTTGGTCTGAC-3′	TEM genotype-R		
Pr441	5′-ATTCTGCTTGGCGCTCCGGG-3′	*adk*-F	583	[39]
Pr442	5′-CCGTCAACTTTCGCGTATTT-3′	*adk*-R		
Pr443	5′-TCACAGGTCGCCAGCGCTTC-3′	*fumC*-F	806	[39]
Pr444	5′-GTACGCAGCGAAAAAGATTC-3′	*fumC*-R		
Pr445	5′-TCGGCGACACGGATGACGGC-3′	*gyrB*-F	911	[39]
Pr446	5′-ATCAGGCCTTCACGCGCATC-3′	*gyrB*-R		
Pr447	5′-ATGGAAAGTAAAGTAGTTGTTCCGGCACA-3′	*icd*-F	878	[39]
Pr448	5′-GGACGCAGCAGGATCTGTT-3′	*icd*-R		
Pr449	5′-CGCGCTGATGAAAGAGATGA-3′	*purA*-F	816	[39]
Pr450	5′-CATACGGTAAGCCACGCAGA-3′	*purA*-R		
Pr451	5′-CGCATTCGCTTTACCCTGACC-3′	*recA*-F	780	[39]
Pr452	5′-TCGTCGAAATCTACGGACCGGA-3′	*recA*-R		
Pr453	5′-ATGAAAGTCGCAGTCCTCGGCGCTGCTGGCGG-3′	*mdh*-F	932	[39]
Pr454	5′-TTAACGAACTCCTGCCCCAGACGCATATCTTTCTT-3′	*mdh*-R		

### Multilocus sequencing typing (MLST)

The extracted DNA described above under ‘Antimicrobial susceptibility testing’ was amplified by PCR using PrimeSTAR GXL DNA polymerase (Takara) with gene-specific primers (Table 1), and sequenced (Macrogen). MLST analyses for *E*. *coli* isolates was performed according to the protocol described in the MLST database for *E*. *coli* (https://enterobase.warwick.ac.uk/species/ecoli/allele_st_search) [40].

### Pulsed-field gel electrophoresis (PFGE)

PFGE was performed as described previously [41], with slight modifications. Briefly, the isolates were cultured in TSB supplemented with 1 μg/ml cefotaxime at 37°C overnight. After centrifugation, PFGE plugs were prepared by mixing culture pellets with 1% SeaKem Gold Agarose (Lonza, Basel, Switzerland). The plugs were digested with 30 U XbaI at 37°C overnight. The plugs were electrophoresed with a CHEF-DR III system (Biorad, Hercules, CA) in a 1% SeaKem Gold Agarose gel in 0.5× TBE buffer at 14°C and 6 V/cm for 16 h. Switching times were ramped from 2.2 to 54.2 s. Dendrographic analysis of the DNA fragments was performed using FPQuest software (Bio-rad). The *Salmonella* serotype Braenderup H9812 strain provided by Dr. Umeda, Osaka Institute of Public Health, was used as a DNA size standard.

### Whole-genome sequencing (WGS) and data analysis

*E*. *coli* isolates were subjected to WGS using the MinION device (Oxford Nanopore Technologies, Oxford, UK) and the MiSeq system (Illumina, San Diego, CA) as described previously [42]. The bacterial isolates were cultured overnight in BHI broth supplemented with 1.28 μg/ml cefotaxime. Genomic DNA was extracted from the cell culture using the DNeasy PowerSoil kit (Qiagen). To prepare MinION sequencing libraries, 1.5–2.5 μg of DNA was sheared using a g-TUBE (Covaris, Woburn, MA) to obtain 8000-bp fragments. One microgram of the sheared DNA was converted to each library using the Ligation Sequencing kit (SQK-LSK109; Oxford Nanopore Technologies) and the Native Barcoding kit (EXP-NBD104; Oxford Nanopore Technologies). To prepare the MiSeq sequencing libraries, Illumina libraries was constructed using the Nextera DNA Flex Library Prep kit (Illumina). Paired-end sequencing was carried out on the MiSeq instrument (Illumina) with the MiSeq Reagent kit version 2 (500 cycles) (Illumina). To construct genomes, a hybrid assembly was conducted using the Unicycler assembler version 0.4.4 [43] and the Flye assembler version 2.6 [44], with sequence reads obtained from the MinION device and the MiSeq instrument. For Flye assembly, assembled sequences were polished using BWA version 0.7.17-r1188 [45] and Pilon version 1.23 [46], with MiSeq sequence reads at three times. Annotation was performed using both the RAST server [47] and the DFAST server [48], followed by manual curation using blastp (https://blast.ncbi.nlm.nih.gov/Blast.cgi). BacWGSTdb 2.0 [49] was used to track the source of whole-genome sequenced isolates. Plasmid replicon typing, identification of resistance and virulence genes and O, H, and FimH typing were carried out using PlasmidFinder 1.3 [50], pMLST 2.0 [50], ResFinder 2.1 [51], VirulenceFinder 2.0 [52], SerotypeFinder [53], and FimTyper [54], respectively. Genetic structures were compared in Easyfig [55].

### SNP calling and phylogenetic analysis

Mobile genetic elements (miniature inverted repeats, insertion sequences, composite transposons, pseudo-composite transposons, unit transposons, integrative conjugative elements, integrative mobilizable elements, and cis-mobilizable elements), prophage, and plasmid regions of the genomic sequences of the reference isolate (described below under each result) were predicted by MGEFinder 1.0.2 [56], PHASTER [57], and PlasmidFinder 1.3, respectively, followed by removal of these regions from the sequences. CSI Phylogeny 1.4 [58] was used to call SNPs and to construct a maximum likelihood phylogenetic tree of *E*. *coli* isolates inferred from the concatenated alignment of detected SNPs. CSI Phylogeny pipeline was run with default parameters: a minimal depth at SNP positions of 10 reads, a minimal relative depth at SNP positions of 10%, a minimal distance between SNPs of 10bp, a minimal Z-score of 1.96, a minimal SNP quality of 30, and a minimal read mapping quality of 25 [59]. The sequence data of an isolate belonging to a different ST were used as the outgroup.

### Conjugal transfer of plasmids

Bacterial conjugation was carried out as described previously [60] with slight modifications. The recipient was a rifampicin-resistant *E*. *coli* K-12 DH5α strain [61] provided by Dr. Umeda, Osaka Institute of Public Health. Overnight cultures of *E*. *coli* isolates in TSB supplemented with 4 μg/ml cefotaxime were mixed with the cultures of the recipient in TSB supplemented with 50 μg/ml rifampicin at a 1:10 ratio. The bacterial mixture was then cultured in tenfold dilutions of TSB overnight without agitation. Transconjugants were selected on TSB agar supplemented with 4 μg/ml cefotaxime and 50 μg/ml rifampicin. The transformation efficiency was calculated as the ratio of the number of transconjugant to recipient colonies. Transfer of the target plasmid was confirmed by testing antimicrobial susceptibility, and by detecting resistance genes in the transconjugants.

### Accession numbers

WGS data were deposited at DDBJ/EMBL/GenBank under the accession numbers BNIP00000000, BNIQ00000000, BNIR00000000, BNIS00000000, BNIT00000000, BNIU00000000, BNIV00000000, BNIW00000000, BNIX00000000, BNIY00000000, BNIZ00000000, BNJA00000000, BNJB00000000, and BNJC00000000.

## Results and discussion

### Prevalence of 3GCR Enterobacteriaceae in companion dogs

The prevalence of concerning AMR Enterobacteriaceae, from a human public-health perspective (carbapenem-resistant, 3GCR, and fluoroquinolone-resistant (FQR) Enterobacteriaceae) [18,19,62], was investigated in 188 extraintestinal bacteria isolated from 138 companion animals with suspected bacterial infectious disease at the Veterinary Medical Center of Osaka Prefecture University. Three, seven, and nine of the 65 Enterobacteriaceae isolates were suspected to be 3GCR, FQR, and both 3GCR and FQR, respectively, by antimicrobial susceptibility tests performed by the outsourcing service. Carbapenem-resistant Enterobacteriaceae were not detected in this study. These results agree with surveillance reports [18,19], which describe the prevalence of 3GCR and FQR Enterobacteriaceae in companion dogs and cats in Japan. The 12 Enterobacteriaceae isolates suspected of being 3GCR were isolated from extraintestinal sites of companion dogs: urinary bladder, skin abscess, abdominal cavity, nasal cavity, or vagina. At the time, these isolates were not defined as extraintestinal pathogenic Enterobacteriaceae, because commensal Enterobacteriaceae can cause extraintestinal infection when the host is compromised [63]. These 12 isolates were obtained from the outsourcing service, and confirmed to be 3GCR by an MIC test (Table 2). Nine of 12 3GCR Enterobacteriaceae isolates were also confirmed to be resistant to fluoroquinolone (IDs: 001Sk, 019S, 019Sk, 024S, 026S, 056S, 066S, 082S, and 123S in Table 2). Fluoroquinolone-resistant *E*. *coli* is very widespread in humans, and FQR is often accompanied by 3GCR, which is mainly caused by the production of ESBLs [64]. For instance, *E*. *coli* ST131 evolved from clade B (FQ sensitive) to clade C (FQR) by chromosomal point mutations in the late 1980s, and then acquired plasmids encoding ESBLs, which led to global domination by this clade [65,66]. Enterobacteriaceae with both FQR and 3GCR were also recently reported in companion animals [67,68]. Given that 75% of tested Enterobacteriaceae isolates (9 of 12) in this study were both FQR and 3GCR, this type of Enterobacteriaceae may have become dominant among 3GCR Enterobacteriaceae in companion animals in Osaka, Japan. The 12 isolates were further analyzed for β-lactamase production by disc diffusion and synergy assays (Table 3). Five isolates (001Sk, 019Sk, 056S, 082S, and 123S) produced ESBLs, and six isolates (019S, 024S, 026S, 066S, 112S, and 128S) were AmpC-producing Enterobacteriaceae. The *Proteus vulgaris* isolate (048Sp) was susceptible to cefotaxime and ceftazidime in the disc diffusion assay, and thus was not characterized for β-lactamase production. All isolates were tested negative for metallo-β-lactamase production.

**Table 2:** MICs of 3GCR Enterobacteriaceae isolated from extraintestinal sites and feces.

ID^1^	Origin	Species	MIC^2^										
			ABPC	CEZ	CMZ	CTX	CAZ	CTRX	CPR	IPM	MEPM	LVFX	CPFX
001Sk	Urine	*K*. *pneumoniae*	>128	>128	0.5	>128	4	>128	64	0.25	1	8	16
001Fk	Feces	*K*. *pneumoniae*	>128	>128	0.5	128	>128	>128	64	0.25	0.12	8	16
019S	Abscess	*E*. *coli*	>128	>128	64	16	64	64	2	0.5	0.12	8	16
019Sk	Abscess	*K*. *pneumoniae*	>128	>128	2	>128	>128	>128	>128	≤0.12	0.12	16	64
019F	Feces	*E*. *coli*	>128	>128	32	16	64	128	1	1	0.12	8	16
019Fk	Feces	*K*. *pneumoniae*	>128	>128	32	>128	>128	>128	>128	0.25	0.5	64	128
024S	Ascites	*E*. *coli*	>128	>128	16	8	16	32	2	0.5	0.12	4	8
024F	Feces	*K*. *pneumoniae*	>128	>128	2	>128	>128	>128	>128	0.25	0.12	32	32
026S	Urine	*E*. *coli*	>128	>128	128	64	128	128	2	0.25	0.12	16	32
026F1	Feces	*E*. *coli*-1	>128	>128	2	>128	>128	>128	>128	≤0.12	0.12	8	32
026F2	Feces	*E*. *coli*-2	>128	>128	128	>128	>128	>128	128	0.5	0.12	16	32
026F3	Feces	*E*. *coli*-3	>128	>128	>128	64	128	64	2	0.5	0.12	16	32
048Sp	Abscess	*P*. *vulgaris*	>128	>128	1	0.12	64	128	0.25	2	0.12	≤0.12	≤0.12
048F	Feces	*E*. *coli*	>128	>128	4	>128	>128	>128	>128	≤0.12	0.12	8	32
049Sm	Urine^3^	*Morganella morganii*	16	>128	8	0.12	0.12	≤0.12	0.12	4	0.12	≤0.12	≤0.12
049F	Feces	*E*. *coli*	>128	>128	4	>128	>128	>128	>128	0.25	0.12	8	32
056S	Nasal cavity	*E*. *coli*	>128	>128	2	>128	>128	>128	>128	≤0.12	0.12	8	32
	Feces	Not provided											
066S	Urine	*E*. *coli*	>128	>128	64	16	64	64	4	0.25	0.12	8	16
066F	Feces	*E*. *coli*	>128	>128	>128	64	>128	>128	16	1	0.12	8	16
066Fc	Feces	*Cedecea* spp.	>128	>128	64	>128	>128	>128	>128	0.25	0.12	16	128
082S	Urine	*E*. *coli*	>128	>128	2	>128	>128	>128	>128	≤0.12	0.12	8	32
082F	Feces	*E*. *coli*	>128	>128	2	>128	>128	>128	>128	≤0.12	0.12	8	32
112S	Urine	*E*. *coli*	>128	>128	32	8	64	32	1	0.5	0.12	0.5	0.25
	Feces	Not detected											
123S	Urine	*E*. *coli*	>128	>128	4	>128	>128	>128	>128	0.5	0.12	64	128
123F	Feces	*E*. *coli*	>128	>128	2	>128	>128	>128	>128	0.5	0.12	32	128
128S	Vagina	*E*. *coli*	>128	>128	32	8	32	32	1	0.5	0.12	0.25	≤0.12
128F	Feces	*E*. *coli*	>128	>128	2	>128	>128	>128	>128	0.25	0.12	0.25	0.25

^1^S, extraintestinal specimen; F, feces; k, *K*. *pneumoniae*; p, *P*. *vulgaris*; m, *M*. *morganii*; c, *Cedecea* spp.

^2^ABPC, ampicillin; CEZ, cefazolin; CMZ, cefmetazole; CTX, cefotaxime; CAZ, ceftazidime; CTRX, ceftriaxone; CPR, cefpirome; IPM, imipenem; MEPM, meropenem; LVFX, levofloxacin; CPFX, ciprofloxacin.

^3^This isolate was sensitive to third-generation cephalosporins.

**Table 3:** Disc diffusion and synergy test results for 3GCR Enterobateriaecae isolated from extraintestinal sites and feces.

ID^1^	Origin	Species	CTX			CAZ			CMZ		IPM		CAZ	
			–	CVA^2^	APB	–	CVA	APB	–	APB	–	SMA	–	SMA
001Sk	Urine	*K*. *pneumoniae*	12	32	13	20	32	21	28	29	28	27	19	19
001Fk	Feces	*K*. *pneumoniae*	10	32	10	20	33	21	28	30	29	27	19	19
019S	Abscess	*E*. *coli*	17	16	21	11	11	19	11	17	23	23	12	11
019Sk	Abscess	*K*. *pneumoniae*	<6	27	10	16	25	17	24	24	26	26	15	15
019F	Feces	*E*. *coli*	14	13	20	7	8	18	9	14	22	22	8	8
019Fk	Feces	*K*. *pneumoniae*	<8	16	<8	10	17	12	8	8	24	24	11	10
024S	Ascites	*E*. *coli*	20	20	24	16	17	22	16	21	25	24	16	16
024Fk	Feces	*K*. *pneumoniae*	<8	23	<8	12	21	13	23	21	27	27	13	13
026S	Urine	*E*. *coli*	14	14	22	10	14	20	8	15	25	25	12	12
026F1	Feces	*E*. *coli*-1	<8	27	11	20	24	19	24	25	25	25	18	18
026F2	Feces	*E*. *coli*-2	<8	17	12	12	14	20	11	17	27	27	12	12
026F3	Feces	*E*. *coli*-3	15	16	20	11	13	21	11	17	22	22	11	11
048Sp	Abscess	*P*. *vulgaris*	28	31	30	28	30	30	25	24	23	22	30	29
048F	Feces	*E*. *coli*	<8	27	<8	18	25	19	23	26	25	25	20	20
049Sm	Urine^3^	*M*. *morganii*	30	35	32	30	30	28	19	20	19	18	30	30
049F	Feces	*E*. *coli*	<8	19	<8	21	22	21	20	21	28	27	23	23
056S	Nasal cavity	*E*. *coli*	<6	28	15	20	25	21	26	29	29	28	20	20
	Feces	Not provided												
066S	Urine	*E*. *coli*	20	20	25	16	17	22	15	19	24	23	14	14
066F	Feces	*E*. *coli*	13	13	19	6	8	19	6	14	22	22	6>	6>
066Fc	Feces	*Cedecea* spp.	<6	10	15	11	12	16	10	14	25	23	7	7
082S	Urine	*E*. *coli*	6	27	22	18	26	20	28	28	27	27	19	19
082F	Feces	*E*. *coli*	10	30	19	18	28	21	27	28	26	26	18	20
112S	Urine	*E*. *coli*	18	18	22	13	12	20	12	17	25	25	12	12
	Feces	Not detected												
123S	Urine	*E*. *coli*	<6	22	<6	7	23	12	25	27	26	26	8	9
123F	Feces	*E*. *coli*	<6	26	13	14	28	19	28	29	27	27	11	12
128S	Vagina	*E*. *coli*	19	18	23	14	13	22	12	20	25	25	13	13
128F	Feces	*E*. *coli*	<6	19	13	12	18	17	14	18	25	25	9	10

^1^S, extraintestinal specimen; F, feces; k, *K*. *pneumoniae*; p, *P*. *vulgaris*; m, *M*. *morganii*; c, *Cedecea* spp.

^2^CVA, clavulanic acid; APB, aminophenylboronic acid; SMA, sodium mercaptoacetate.

^3^This isolate was sensitive to third-generation cephalosporins.

Dog feces were then examined for the presence of the ESBL/AmpC-producing Enterobacteriaceae. Fourteen 3GCR Enterobacteriaceae isolates were isolated from 12 dogs (Table 2). Nine of 14 isolates (001Fk, 019Fk, 024F, 026F1, 026F2, 048F, 049F, 082F, and 123F) were ESBL-producing *E*. *coli* or *K*. *pneumoniae*. Three isolates (019F, 026F3, and 066F) were AmpC-producing *E*. *coli*. Two isolates (066Fc and 128F) were both ESBL- and AmpC-producing Enterobacteriaceae (Table 3). These results showed that 81.8% (9 of 11) of the tested companion dogs harbored ESBL/AmpC-producing bacteria in their intestinal tracts.

### WGS-based characterization of ESBL/AmpC-producing *E*. *coli* isolates from companion dogs

We focused on 19 *E*. *coli* isolates from the extraintestinal sites and feces of 11 dogs (S1 Fig). These isolates belonged to seven STs: ST131 (n = 6), ST162 (n = 5), ST68 (n = 2), ST405 (n = 2), ST998 (n = 2), ST10 (n = 1), and ST38 (n = 1). In addition, the isolates harbored one, two, or three copies of ESBL/AmpC genes encoding TEM-1A (n = 4), TEM-1B (n = 4), TEM-1C (n = 1), CTX-M-14 (n = 1), CTX-M-15 (n = 3), CTX-M-27 (n = 6), and CMY-2 (n = 10). To address whether *E*. *coli* isolates from companion dogs can infect humans, 14 *E*. *coli* isolates were whole-genome sequenced (Tables 4, 5 and S1), and their genomic similarities with strains isolated from humans were evaluated.

**Table 4:** WGS for 14 strains, with particular respect to resistance genes^1^.

ID^2^	MLST	Type			Circular^3^	Size (bp)^4^		Plasmid replicon type	pMLST	Resistance gene	
		O	H	FimH						Insertion	Point mutation
019S	162	9	9	32	Y	Ch	4,786,997			*mdf(A)/aac(3)-IId/bla*_TEM-1B_	*gyrA*(S83L, D87N)/*parC*(S80I)
					Y	p3	71,894	IncFII	F40:A−:B−	*bla*_CMY-2_	
024S	162	9	9	32	N		4,863,954	IncI1		*mdf(A)/bla*_CMY-2_*/sul2/aph(3’’)-Ib/aph(6)-Id*	*gyrA*(S83L, D87N)/*parC*(S80I)
026S	68	NT^5^	6	49	Y	Ch	5,228,405			*mdf(A)*	*gyrA*(S83L, D87N)/*parC*(S80I)
026F2	131	25	4	30	Y	Ch	5,022,176			*mdf(A)*	*gyrA*(S83L, D87N)/*parC*(S80I, E84V)/*parE*(I529L)/
					Y	p2	132,883	IncFIA/FIB/FII	F1:A2:B20	*dfrA17/aadA5/sul1/mph(A)/sul2/aph(3’’)-Ib/aph(6)-Id/tet(A)/bla*_CTX-M-27_	
026F3	68	NT	6	49	Y	Ch	5,228,549			*mdf(A)*	*gyrA*(S83L, D87N)/*parC*(S80I)/*pmrB*(V161G)
					N	c3	83,620	IncI1	UT^6^	*bla*_CMY-2_	
048F	131	25	4	30	Y	Ch	4,976,521			*mdf(A)*	*gyrA*(S83L, D87N)/*parC*(S80I, E84V)/*parE*(I529L)/
					Y	p2	116,719	IncFIA/FIB/FII	F1:A2:B20	*dfrA17/aadA5/sul1/mph(A)/sul2/aph(3’’)-Ib/aph(6)-Id/tet(A)/bla*_CTX-M-27_	
049F	38	86	18	5	Y	Ch	4,941,596			*mdf(A)/bla*_CTX-M-14_*/bla*_CTX-M-14_	*gyrA*(S83L, D87Y)/*parC*(S80I)/*parE*(S458A)
056S	131	25	4	30	Y	Ch	5,036,476			*mdf(A)*	*gyrA*(S83L, D87N)/*parC*(S80I, E84V)/*parE*(I529L)
					Y	p3	81,700	IncFIA/FIB/FII	F1:A2:B20	*bla*_CTX-M-27_	
066S	162	9	9	32	Y	Ch	4,761,096			*mdf(A)/aac(3)-IId/bla*_TEM-1B_	*gyrA*(S83L, D87N)/*parC*(S80I)
					Y	p3	71,894	IncFII	F40:A−:B−	*bla*_CMY-2_	
082S	131	25	4	30	N		5,095,678			*mdf(A)*	*gyrA*(S83L, D87N)/*parC*(S80I, E84V)/*parE*(I529L)
					Y	p2	120,446	IncFIA/FIB/FII	F1:A2:B20	*bla*_CTX-M-27_	
112S	10	157	16	24	Y	Ch	4,890,648				
					N	c2	104,408	IncI1	12	*bla*_CMY-2_	
					Y	p4	123,831	IncX1	NT	*aph(3’’)-Ib/aph(6)-Id/qnrS1/sul1/sul2/tet(A)/mph(A)*	
					N	c5	57,689	IncFII	F2:A−:B−	*aadA5/dfrA17*	
123S	405	102	5	27	Y	Ch	5,141,049			*mdf(A)*	*gyrA*(S83L, D87N)/*parC*(S80I)
					Y	p4	45,297	ND^7^		*bla*_CTX-M-15_*/bla*_TEM-1B_*/tet(A)/aac(6’)-Ib-cr*	
					Y	p5	117,362	IncFII	K2:A−:B−	*bla*_CTX-M-15_*/bla*_TEM-1C_*/ARR-3/tet(A)/aac(6’)-Ib-cr/aadA16/mph(A)/dfrA27/sul1*	
128S	998	50/2	6	14	Y	Ch	5,242,331			*mdf(A)*	*gyrA*(S83L)/*pmrB*(V161G)
					Y	p2	129,879	IncFIA/FIB/FII	F1:A2:B45	*bla*_TEM-1B_	
					Y	p3	101,235	IncC	3	*bla*_CMY-2_*/tet(A)/aph(3”)-Ib/aph(6)-Id/sul2/mph(A)/floR*	
128F	998	50/2	6	14	Y	Ch	5,244,017			*mdf(A)*	*gyrA*(S83L)/*pmrB*(V161G)
					N	c5	8,211				
					N	c6	82,702	IncI1	16	*bla*_CTX-M-15_*/bla*_TEM-1B_	
					N	c7	101,229	IncC	3	*bla*_CMY-2_*/tet(A)/aph(3”)-Ib/aph(6)-Id/sul2/mph(A)/floR*	

^1^Details are described in S1 Table.

^2^S, extraintestinal specimen; F, feces.

^3^Y, circular; N, not circular.

^4^Ch, chromosome; p, plasmid; c, contig.

^5^Not typed.

^6^Untypable.

^7^Not determined.

**Table 5:** WGS for 14 strains, with particular respect to virulence genes^1^.

ID^2^	MLST	Type			Circular^3^	Size (bp)^4^		Plasmid replicon type	pMLST	Virulence gene
		O	H	FimH						
019S	162	9	9	32	Y	Ch	4,786,997			*fyuA/gad/irp2/iss/lpfA/terC*
					Y	p2	122,754	IncFII	F18:A−:B58	*cvaC/etsC/hlyF/iroN/iss/iucC/iutA/mchF/ompT/sitA/traT*
					Y	p3	71,894	IncFII	F40:A−:B−	*cba/cia/cma/traT*
024S	162	9	9	32	N		4,863,954	IncI1		*fyuA/gad/irp2/iss/lpfA/ompT/terC*
					Y	p2	136,773	IncFII		*cvaC/etsC/hlyF/iroN/iss/iucC/iutA/mchF/ompT/sitA/traT*
026S	68	NT^5^	6	49	Y	Ch	5,228,405			*air/chuA/eilA/fyuA/gad/iha/irp2/iss/iucC/iutA/kpsE/kpsMII/lpfA/papA_F7-2/papC/sat/sitA/terC*
					Y	p2	62,243	IncFII	F104:A−:B−	*traT*
026F2	131	25	4	30	Y	Ch	5,022,176			*chuA/fyuA/gad/iha/irp2/iss/iucC/iutA/kpsE/kpsMII_KS/ompT/papA_F43/sat/sitA/terC/usp/yfcV*
					Y	p2	132,883	IncFII	F1:A2:B20	*senB/traT*
026F3	68	NT	6	49	Y	Ch	5,228,549			*air/chuA/eilA/fyuA/gad/iha/irp2/iss/iucC/iutA/kpsE/kpsMII/lpfA/papA_F7-2/papC/sat/sitA/terC*
					N	c4	56,813	IncFII	F104:A−:B−	*traT*
048F	131	25	4	30	Y	Ch	4,976,521			*chuA/fyuA/gad/iha/irp2/iss/iucC/iutA/kpsE/kpsMII_K5/ompT/papA_F43/sat/sitA/terC/usp/yfcV*
					Y	p2	116,719	IncFII	F1:A2:B20	*senB/traT*
049F	38	86	18	5	Y	Ch	4,941,596			*air/chuA/eilA/fyuA/gad/hra/irp2/iss/kpsE/kpsMII_K5/terC*
					Y	p2	110,583	IncFII	F51:A−:B10	*senB/traT*
					Y	p4	102,731	IncB/O/K/Z		*traT*
					Y	p5	66,287	IncFII	F52:A−:B−	*traT*
056S	131	25	4	30	Y	Ch	5,036,476			*chuA/fyuA/gad/iha/irp2/iss/iucC/iutA/kpsE/kpsMII_K5/ompT/papA_F43/sat/sitA/terC/usp/yfcV*
					Y	p2	86,103			*traT*
					Y	p3	81,700	IncFII	F1:A2:B20	*traT*
					Y	p6	6,077	Col156		*celb*
066S	162	9	9	32	Y	Ch	4,761,096			*fyuA/gad/irp2/lpfA/terC*
					Y	p2	121,066	IncFII	F18:A−:B1	*cvaC/etsC/hlyF/iroN/iss/iucC/iutA/mchF/ompT/sitA/traT*
					Y	p3	71,894	IncFII	F40:A−:B−	*cba/cia/cma/traT*
082S	131	25	4	30	N		5,095,678			*astA/chuA/fyuA/gad/hra/iha/irp2/iss/iucC/iutA/kpsE/kpsMII/ompT/sat/sitA/terC/usp/yfcV*
					Y	p2	120,446	IncFII	F1:A2:B20	*senB*
					Y	p3	72,689	IncFII	F4:A−:B−	*mcbA/traT*
112S	10	157	16	24	Y	Ch	4,890,648			*astA/eae/efa1/espA/espB/espF/espJ/gad/hra/nleA/nleB/nleC/tccP/terC/tir*
					N	c2	104,408	IncI1	12	*cib*
					N	c3	55,667	IncFII		*traT*
					N	c5	57,689	IncFII	F2:A−:B−	*traT*
					Y	p6	58,375	IncFII	F30:A−:B−	*sepA*
123S	405	102	5	27	Y	Ch	5,141,049			*afaA/afaB/afaC/afaD/afaE/air/chuA/eilA/fyuA/gad/irp2/kpsE/kpsMII/sitA/terC*
					Y	p2	94,304	IncFII	F−:A6:B20	*traT*
					Y	p3	94,328			*cia*
					Y	p4	45,297	ND^6^		*mcbA*
					Y	p5	117,362	IncFII	K2:A−:B−	*traT*
128S	998	50/2	6	14	Y	Ch	5,242,331			*chuA/clbB/cnf1/focCsfaE/fyuA/gad/hra/ibeA/ireA/iroN/irp2/iss/kpsE/kpsMII_K1/mchB/mchC/mchF/mcmA/neuC/ompT/papA_F13/papC/pic/sfaD/sfaS/sitA/terC/usp/vat/yfcV*
					Y	p2	129,879	IncFII	F1:A2:B45	*senB/traT*
128F	998	50/2	6	14	Y	Ch	5,244,017			*chuA/clbB/cnf1/focCsfaE/fyuA/gad/hra/ibeA/ireA/iroN/irp2/iss/kpsE/kpsMII_K1/mchB/mchC/mchF/mcmA/neuC/ompT/papA_F13/papC/pic/sfaD/sfaS/sitA/terC/usp/vat/yfcV*
					N	c3	118,353	IncFII	F1:A2:B45	*senB/traT*

^1^Details were described in S1 Table.

^2^S, extraintestinal specimen; F, feces.

^3^Y, circular; N, not circular.

^4^Ch, chromosome; p, plasmid; c, contig.

^5^Not typed.

^6^Not determined.

First, we investigated the genetic relatedness between multiple isolates from companion dogs belonging to the same ST. Two previous studies revealed that maximum SNP counts were four [69] and 15 [70] for the same outbreak of *E*. *coli* foodborne illness. To determine if multiple isolates from companion dogs with the same ST arise from the same source (belong to the same outbreak event), pairwise SNP differences were measured using core genome regions from whole-genome sequences (S2 Table). Six and eight SNP differences were identified for ST68 isolates (026S and 026F3) and ST998 isolates (128S and 128F), respectively. In both ST isolates, one isolate (026S or 128S) was isolated from the extraintestinal site and the other (026F3 or 128F) was from the feces of the same dog. These results strongly suggest that these two isolates in ST68 and ST998, respectively, arose from the same source. PFGE and the resulting dendrogram also revealed that each *E*. *coli* isolate from the extraintestinal site had the closest genetic relationship with the isolate from feces of the same dog (>85% pattern similarity) (S1 Fig). Intestinal colonization by human extraintestinal pathogenic *E*. *coli* (ExPEC) was demonstrated in companion dogs [71]. Furthermore, closely related or indistinguishable ExPEC strains (by typing methods) have been recovered from humans and their companion animals [72,73]. Taken together, these results suggest that canine extraintestinal ESBL/AmpC-producing *E*. *coli* can spread via feces, which may lead to cross-transmission to humans. Four ST131 isolates carried 77–178 SNPs and were not likely to belong to the same outbreak event (S2 Table). In ST162 isolates, three SNP differences were identified between the isolates 019S and 066S, which were derived from different companion dogs (S2 Table). These results strongly suggest that these two isolates arose from the same source (possibly from the same outbreak).

The closely related isolates were then predicted using the genome-based SNP strategy of the single genome analysis tool on the BacWGST database to track the source of the whole-genome sequenced 14 isolates from companion dogs (S3 Table). Isolates 026F2, 048F, 049F, 056S, 082S, 112S, and 123S had the closely related isolates on the BacWGST database. SNP differences ranged from 454–947 between the isolates (049F, 112S, and 123S) and their closest isolates. The presence of many (hundreds or more) SNPs indicates that the isolates were distantly related, implying that they did not originate from the same reservoir population [74]. Therefore, the sources of isolates 049F, 112S, and 123S could not be tracked or identified. Each ST131 isolate (026F2, 048F, 056S, and 082S) differed from its closest isolate by 80–247 SNPs. All the closest isolates were isolated from infection sites in human and were likely to be human ExPECs. These results suggested that ST131 isolates from companion dogs arose from human ExPECs although we were unable to identify the recent ancestor of ST131 isolates because of the high SNP differences.

Next, we focused on AMR-associated genes. Four isolates (026F2, 048F, 056S, and 082S) harbored *bla*_CTX-M-27_ in IncF incompatibility group plasmids with the pMLST type F1:A2:B20. These isolates all belonged to the ST131 and O25:H4:FimH30 types. Two isolates (123S and 128F) harbored *bla*_CTX-M-15_ in plasmids. The ST38 isolate, 049F, possessed two *bla*_CTX-M-14_ genes on its chromosome. Seven isolates (019S, 024S, 026F3, 066S, 112S, 128S, and 128F) harbored *bla*_CMY-2_, six of which harbored the gene in plasmids. We could not determine whether the *bla*_CMY-2_ gene of isolate 024S was located on its chromosome or on a plasmid, because we did not successfully determine the whole chromosomal sequence. No β-lactamase genes were detected from isolate 026S in our analysis. All the isolates, except 112S, had point mutations in quinolone resistance-determining regions of the protein. Resistance to fluoroquinolone requires the concurrent presence of at least three mutations in the target proteins encoded on the chromosome; the most common are amino acid changes in positions S83 and D87 of GyrA and S80 of ParC [75]. Two isolates, 128S and 128F, showed only one point mutation (S83L in GyrA) and also lacked the plasmid-borne quinolone resistance-associated genes *qnr* and *aac*(6*’*)-*Ib*-*cr*, resulting in quinolone susceptibility. In contrast, the remaining isolates had at least three mutations as described above, leading to quinolone resistance (Tables 2 and 4).

#### (i) *bla*_CTX-M-27_ in the ST131 lineage

**Fig 1:**
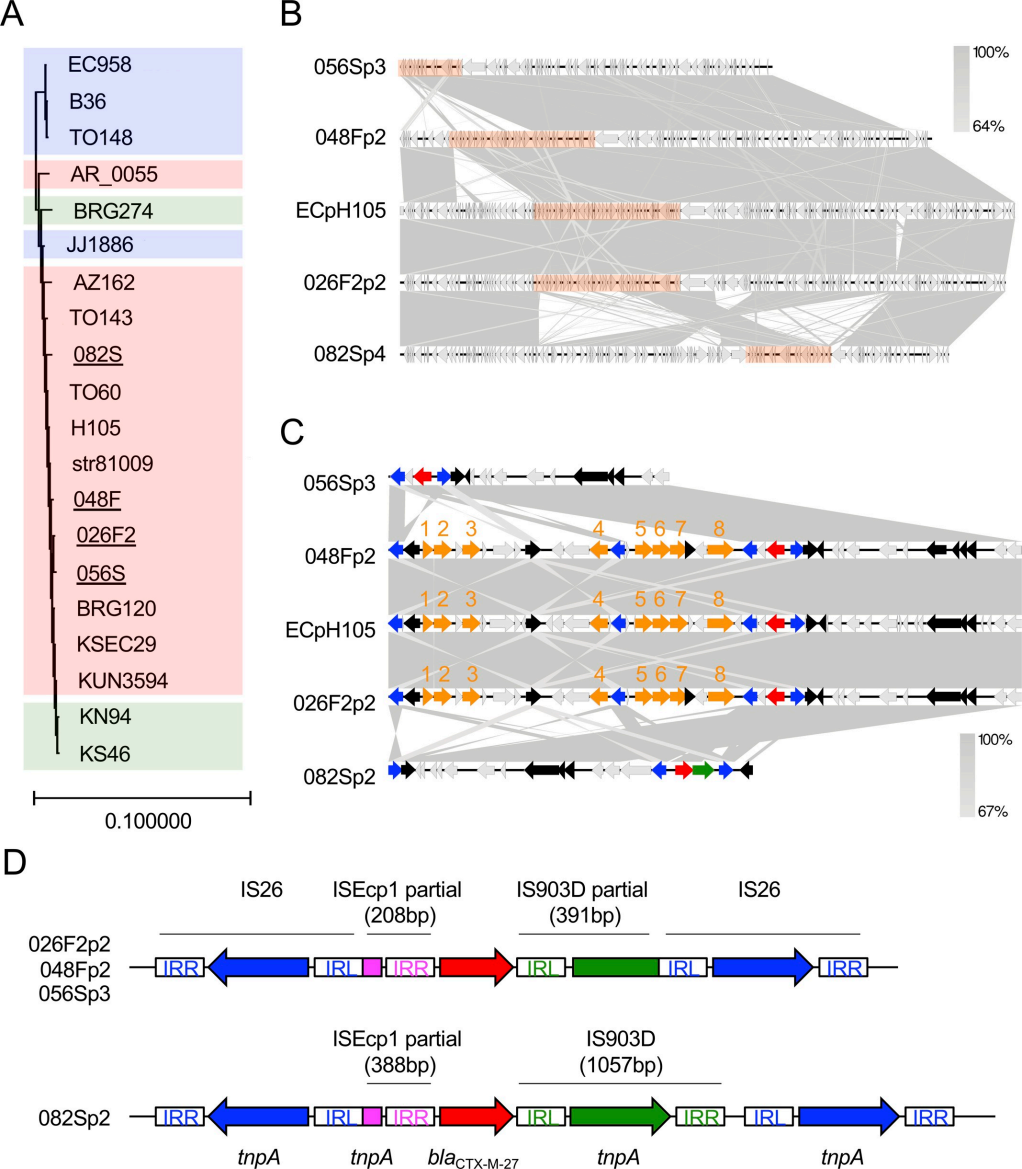
Characteristics of ESBL-producing *E*. *coli* ST131 isolates. (A) A maximum likelihood chromosomal phylogenetic tree based on core-genome wide SNPs depicts four ESBL-producing *E*. *coli* isolates of the ST131 lineage C1-M27 from companion dogs (underlined) along with those from the human-derived ST131 lineage C1-M27 (red), clade C1/*H*30R harboring *bla*_CTX-M-14_ (green), and clade C2/*H*30Rx harboring *bla*_CTX-M-15_ (blue), which were extracted from the NCBI database. *E*. *coli* JJ1886 was used as a reference strain. The chromosomal sequence of the ST998 isolate 128S in this study was used as the outgroup. (B) The *bla*_CTX-M-27_-coding plasmids isolated in this study were compared with a highly similar plasmid, pH105, from *E*. *coli* strain 105. Homologous regions are shaded gray. The orange bars are enlarged in Fig 1C. (C) An enlargement of partial sequences of the *bla*_CTX-M-27_-coding plasmid depicted in Fig 1B. Genetic elements are indicated by colors as follows: *bla*_CTX-M-27_, red; other antimicrobial resistance genes, orange; IS26 *tnpA*, blue; IS903D *tnpA*, green; other mobile genetic elements, black. Genes are indicated by numbers as follows: *dfrA17*, 1; *aadA5*, 2; *sul1*, 3; *mph(A)*, 4; *sul2*, 5; *aph(3′′)-Ib*, 6; *aph(6)-Id*, 7; *tetA*, 8. (D) Details of the *bla*_CTX-M-27_ transposon elements of 026F2p2, 048Fp2, 056Sp3 (upper panel), and 082Sp2 (lower panel). IRL and IRR are inverted repeats of each insertion sequence. Nucleotide sequences of each IR are as follows: IS26-IRL, GGCACTGTTGCAAA; IS26-IRR, TTTGCAACAGTGCC; IS903D-IRL, GGTTTTGTTGAATAAATC; IS903D-IRR, GATTTATTCAACAAAGCC; ISEcp1-IRR, ACTGTTAATTTAGG. Accession numbers of human-derived ST131 isolates and plasmids are as follows: JJ1886, PRJNA218163; TO148, PRJEB27474; EC958, PRJNA283246; B36, PRJEB29930; AZ162, SAMN06187728; AR_0055, PRJNA292904; TO143, PRJEB27473; TO60, PRJEB27477; str81009, PRJNA383781; H105, PRJNA387731; BRG120, SAMD00044968; KUN3594, SAMD00044940; KSEC29, SAMD00044934; BRG274, SAMD00044971; KN94, SAMD00044963; KS46, SAMD00044955; ECpH105, CP021871.1.

Four isolates (026F2, 048F, 056S, and 082S) were of the ST131 and O25:H4:FimH30 type. These isolates all harbored *bla*_CTX-M-27_ in multireplicon plasmid depicting IncFIA/FIB/FII replicons with the pMLST type F1:A2:B20, and acquired fluoroquinolone resistance by point mutations in *parC* (S80I and E84V), *parE* (I529L), and *gyrA* (S83L and D87N). These results indicate that these four isolates belong to the C1-M27 clade within the C1/*H*30R clade [65,66]. In 2006, C1-M27 isolates of the ST131 lineage were detected in humans in Japan, and the numbers of this clade escalated in the 2010s [76]. Recently, this clade emerged and spread in France and Germany [77,78]. These reports showed that the ST131/C1-M27 clade is responsible for the substantial increase in ESBL-producing ExPEC in humans. We considered whether C1-M27 isolates could be transmitted between dogs and humans, and therefore evaluated genomic similarities between dog and human isolates. The chromosomal sequences of four C1-M27 isolates from companion dogs were compared with those from the human-derived ST131 lineage C1-M27, the clade C1/*H*30R harboring *bla*_CTX-M-14_, and the clade C2/*H*30Rx harboring *bla*_CTX-M-15_ [78,79], extracted from the NCBI database. A phylogenetic analysis based on genome-wide SNPs revealed that isolates in this study (underlined in Fig 1A) were highly similar to the human-derived C1-M27 clade (colored red in Fig 1A). In particular, *E*. *coli* str81009 (a human ExPEC isolated from human urine in UAE in 2009) carried 23–36 SNP differences against the isolates 026F2, 048F, and 056S from companion dogs (S4 Table). These SNP differences (23–36) between *E*. *coli* str81009 and the isolates 026F2, 048F, and 056S were lower than those among companion dogs (48–61 SNPs). Taken together, the results suggest that each of the three isolates 026F2, 048F, and 056S arose from human ExPEC and then evolved independently. We next focused on the IncF/pMLST F1:A2:B20 plasmid harboring *bla*_CTX-M-27_. The plasmid from 026F2 showed 99.2% nucleotide identity and 100% query coverage with the human-derived pH105 plasmid by blastn search (Fig 1B). pH105 originated from a human-derived *E*. *coli* H105 strain that was isolated in Germany in 2010, the chromosome of which shared high similarity with that of isolates 026F2, 048F, and 056S in Fig 1A and S4 Table. Two of the four plasmids (plasmid 2 of 026F2: 026F2p2 and 048Fp2) had genes conferring resistance to aminoglycosides *(aadA5*, *aph(3′′)-1b*, and *aph(6)-1d*), macrolides (*mph(A)*), tetracycline (*tetA*), sulfonamide (*sul1* and *sul2*), and trimethoprim (*dfrA17*), which were identical to those in pH105 (Fig 1C). All the plasmids had an IS26-ΔISEcp1-*bla*_CTX-M-27_-ΔIS903D/IS903D-IS26-like structure [80–82] (Fig 1D). The structures shared 100% identity in 026F2p2, 048Fp2, and 056Sp3, and were common in the human C1-M27 clade [76,83]. By contrast, the 082Sp2 plasmid contained an intact IS903D element between *bla*_CTX-M-27_ and IS26 that appeared to be unique, because there is no report describing an IS26-ΔISEcp1-*bla*_CTX-M-27_-IS903D-IS26 structure to the best of our knowledge. Conjugative transfer assays revealed that these four F1:A2:B20 plasmids harboring *bla*_CTX-M-27_ could be transmitted horizontally (Table 6). These results strongly suggest that the C1-M27 clade of the ST131 lineage is transmitted between companion dogs and humans, and also that the F1:A2:B20 plasmid with *bla*_CTX-M-27_ can be transferred between strains colonizing the two host species.

**Table 6:** Summary of conjugal assay results.

ID	Conjugation frequency^1^	CTX^2^			CAZ^2^			CMZ^2^	
		–	CVA	APB	–	CVA	APB	–	APB
w/o^3^		48	ND^4^	ND	41	ND	ND	41	ND
019S	(7.76 ± 0.13)×10^−5^	20	18	23	16	14	23	16	19
024S	(1.99 ± 1.43)×10^−6^	18	15	23	13	14	21	13	19
026F2	(7.11 ± 7.23)×10^−2^	<6	26	17	20	26	20	27	28
026F3	(4.22 ± 3.57)×10^−6^	7	11	20	7	7	20	6	12
048F	(1.80 ± 1.40)×10^−5^	7	27	23	19	26	22	26	26
056S	(9.41 ± 9.01)×10^−4^	7	30	22	20	28	21	30	30
066S	(4.13 ± 6.47)×10^−5^	14	12	22	12	11	21	<6	20
082S	(3.43 ± 2.87)×10^−3^	<6	32	14	16	36	20	30	32
112S	(2.86 ± 4.77)×10^−2^	17	16	26	15	12	26	6	18
123S	(5.59 ± 1.71)×10^−1^	<6	22	<6	6	24	11	25	28
128S	(3.34 ± 2.92)×10^−6^	11	11	23	8	9	23	8	15
128F	(3.07 ± 2.39)×10^−4^	<6	20	18	15	16	19	15	20

^1^Data represent the mean ± SD from three independent experiments.

^2^Disc diffusion assay results of transconjugants.

^3^Disc diffusion assay results of the recipient *E*. *coli* strain.

^4^Not determined.

#### (ii) Plasmids harboring the *bla*_CTX-M-15_ gene

**Fig 2:**
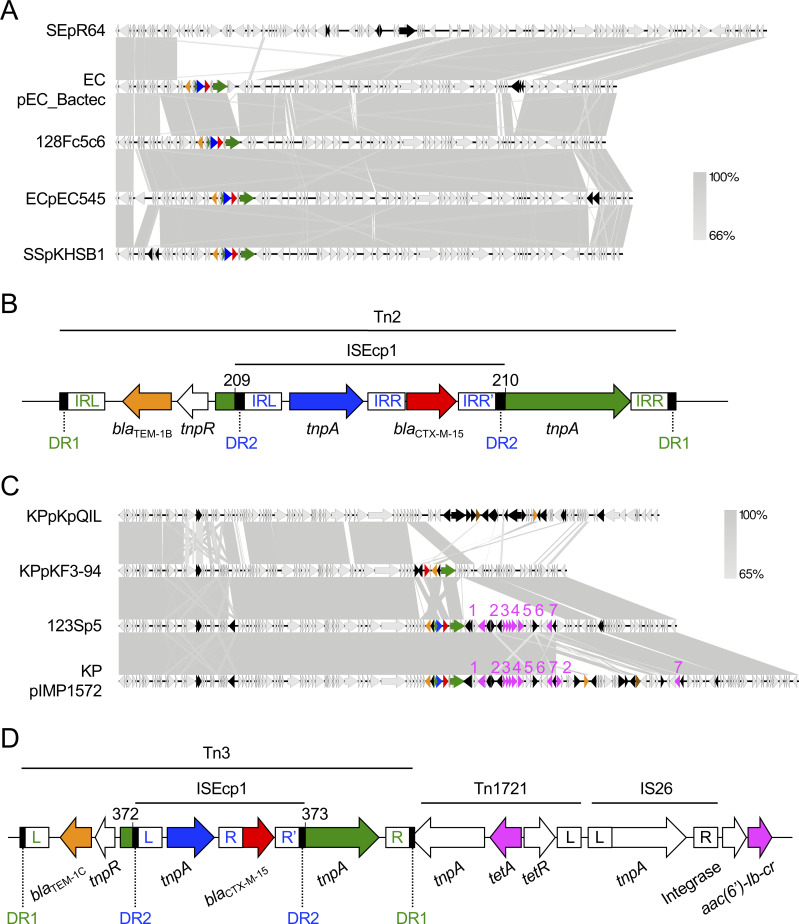
Characteristics of *bla*_CTX-M-15_-coding plasmids. (A) IncI1/pMLST16 contig 128Fc5c6 coding *bla*_CTX-M-15_ was compared with a prototype or the highly homologous plasmids pR64 from *S*. *enterica*, pEC_Bactec from *E*. *coli*, pEC545 from *E*. *coli*, and pKHSB1 from *S*. *sonnei*. Homologous regions are shaded gray. Genetic elements are indicated by colors as follows: *bla*_CTX-M-15_, red; *bla*_TEM-1_, orange; ISEcp1 *tnpA*, blue; Tn2 *tnpA*, green; other mobile genetic elements, black. (B) Details of the *bla*_CTX-M-15_ transposon element of 128Fc5c6 in Fig 2A. The basepair numbering corresponds to segments of the Tn2 *tnpA* gene that were separated by the transposon. DR1 and DR2 indicate the 5-bp direct repeats for Tn2 (GAAAA) and ISEcp1 (TCATA), respectively. IRs in green and blue indicate the inverted repeats for Tn2 and ISEcp1, respectively. (C) The *bla*_CTX-M-15_-coding IncFII_K_/K2:A−:B− plasmid 123Sp5 was compared with pKpQIL, pKF3-94, and pIMP1572 from *K*. *pneumoniae* isolates that shared highly homology with 123Sp5. Genes are indicated by colors as follows: *bla*_CTX-M-15_, red; *bla*_TEM-1_, orange; *bla*_IMP_ or *bla*_KPC_, brown; other antimicrobial resistance genes, pink; ISEcp1 *tnpA*, blue; Tn3 *tnpA*, green; other mobile genetic elements, black. Genes are indicated by numbers as follows: *tetA*, 1; *aac(6′)-Ib-cr*, 2; *ARR-3*, 3; *dfrA27*, 4; *aadA16*, 5; *sul1*, 6; *mph(A)*, 7. (D) Details of the *bla*_CTX-M-15_ transposon element of 123Sp5 in Fig 2C. The basepair numbering corresponds to segments of the Tn3 *tnpA* gene that were separated by the transposon. DR1 and DR2 indicate the 5-bp direct repeats for Tn3 (GTTAA) and ISEcp1 (TCATA), respectively. L, R, and R’ for the mobile elements are the left, right, and alternative right IRs, respectively. Nucleotide sequences of each IR are as follows: Tn2 and Tn3-IRL, GGGGTCTGACGCTCAGTGGAACGAAAACTCACGTTAAG; Tn2 and Tn3-IRR, CTTAACGTGAGTTTTCGTTCCACTGAGCGTCAGACCCC; ISEcp1-IRL, CCTAGATTCTACGT; ISEcp1-IRR, ACGTGGAATTTAGG; ISEcp1-IRR’, ACGTAGGTCCCAGG; Tn1721-IRL, GGGGAGCCCGCAGAATTCGGAAAAAATCGTACGCTAAG; IS26-IRL, GGCACTGTTGCAAA; IS26-IRR, TTTGCAACAGTGCC. Accession numbers of database-derived plasmids are as follows: pR64, AP005147; pEC_Bactec, GU371927; pEC545, CP018975; pKHSB1, HF572032; pKpQIL, GU595196; pKF3-94, FJ876826; pIMP1572, MH464586.

Two isolates (123S and 128F) harbored the plasmid coding for *bla*_CTX-M-15_. We failed to determine the whole plasmid sequence in 128F, but analysis of contigs c5 and c6 of 128F (128Fc5c6) revealed that it belonged to the incompatibility group IncI1/pMLST16, and carried two ESBL genes, *bla*_CTX-M-15_ and *bla*_TEM-1B_. This plasmid showed high similarity (97.9% identity, 95.0% query coverage) to the previously published plasmid pEC_Bactec from a horse-derived *E*. *coli* strain that was isolated in 2010 [84] (Fig 2A). In comparison with the reference plasmid for the IncI1 group pR64 from *Salmonella enterica*, pEC_Bactec arose by transposition of Tn2 carrying *bla*_TEM_ and ISEcp1-*bla*_CTX-M-15_ [84]. 128Fc5c6 had a conserved transposon–ESBL gene element (Fig 2B) [85–87]. The *bla*_TEM-1B_ gene was located in a Tn2-like transposon that possessed intact 38-bp inverted repeats (IR, colored in green in Fig 2B). The Tn2-like transposon was flanked by 5-bp direct repeats (DR1 in Fig 2B). The *tnpA* gene of the Tn2 transposon was disrupted between nucleotides 209 and 210 by transposition of the ISEcp1-*bla*_CTX-M-15_ element. ISEcp1-mediated transposition created a 5-bp duplication of the target sequence (DR2 in Fig 2B). This mechanism involved the left IR (IRL) of ISEcp1, and a right alternative IR (IRR’) that resembled the IRR of ISEcp1 (IR colored in blue in Fig 2B). The plasmid shared more than 95% homology with pEC545 from a human-derived *E*. *coli* isolate (from Vietnam in 2017) and pKHSB1 from human-derived *Shigella sonnei*, which was prevalent among humans in Vietnam and Korea [88,89] (Fig 2A). IncI1/pMLST16 harboring *bla*_CTX-M-15_ was found in various bacterial species, continents, and host species; for instance, human-derived *E*. *coli* isolates in Ireland [90], cattle-derived *E*. *coli* isolates in the UK [90], human-derived *S*. *enterica* isolates [91], and the human-derived *S*. *sonnei* isolates in Vietnam and Korea described above [89]. Given that the plasmid containing 128Fc5c6 was capable of horizontal transfer (Table 6), we strongly suggest that this type of plasmid is shared between humans and companion dogs. The plasmids have spread in several continents, hosted by both zoonotic pathogens and commensal bacterial species, circulating among both humans and animals. Companion dogs might contribute to the spread of this type of plasmid among humans.

The other plasmid, 123Sp5, belonged to the incompatibility group IncFII_K_ with the pMLST type K2:A−:B−. This replicon plasmid type originated from *K*. *pneumoniae* [92,93] and was found in several *E*. *coli* isolates [94], indicating that IncFII_K_/K2:A−:B− can transfer from *K*. *pneumoniae* to other bacterial species. Conjugal assays in this study revealed that 123Sp5 was capable of horizontal transfer (Table 6), suggesting that 123Sp5 can be transmitted between these two bacterial species. Indeed, 123Sp5 shared high homology with plasmids from *K*. *pneumoniae*: IncFII_K_ early plasmid pKF3-94 [95] and pIMP1572 [96] (nucleotide identity, 99.97%; query coverage, 79% and 98%, respectively), and all the plasmids harbored *bla*_CTX-M-15_ (Fig 2C). pKpQIL, an IncFII_K2_ (K2:A−:B−) group plasmid, is one of the most common *bla*_KPC_-harboring plasmids [94,97]. 123Sp5 shared less homology (96.81% identity and 51% query coverage) with pKpQIL than it did with pKF3-94 and pIMP1572, and did not possess the carbapenem-resistance gene. 123Sp5 was closely related to pKF3-94, but possessed genes conferring multidrug resistance to aminoglycosides (*aadA16*), trimethoprim (*dfrA27*), fluoroquinolones (*aac(6cl-Ib-cr*), tetracycline (*tetA*), macrolides (*mph(A)*), and sulfonamide (*sul1*), in addition to the EBSL genes. The *bla*_TEM-1C_ gene was located in a Tn3-like transposon. The Tn3 *tnpA* gene was disrupted between nucleotides 372 and 373 by the ISEcp1-*bla*_CTX-M-15_ element [84–87] (Fig 2D). This structure was highly similar to that in 128Fc5c6 (Fig 2B). Furthermore, Tn1721 *tnpA* composing the *tetA* mobile element was disrupted by the Tn3 transposon element carrying *bla*_TEM-1C_ and ISEcp1-*bla*_CTX-M-15_ (Fig 2D). This indicates that *bla*_TEM-1C_ and *bla*_CTX-M-15_ were inserted after the *tetA* element was translocated; and therefore, pKF3-94 did not appear to be the direct ancestor of 123Sp5. Another closely related plasmid, pIMP1572, additionally possessed *bla*_IMP_, potentially leading to carbapenem resistance. In this study, it was not clear whether the 123Sp5-like plasmid or *E*. *coli* carrying a 123Sp5-like plasmid was transmitted between humans and companion dogs. However, the prevalence of this type of plasmid should be longitudinally monitored, because mobile elements may be easily inserted or deleted on the locus coding for multidrug-resistance genes, and the plasmid can be transmitted horizontally outside of the bacterial species.

#### (iii) Chromosomal insertion of *bla*_CTX-M-14_

**Fig 3:**
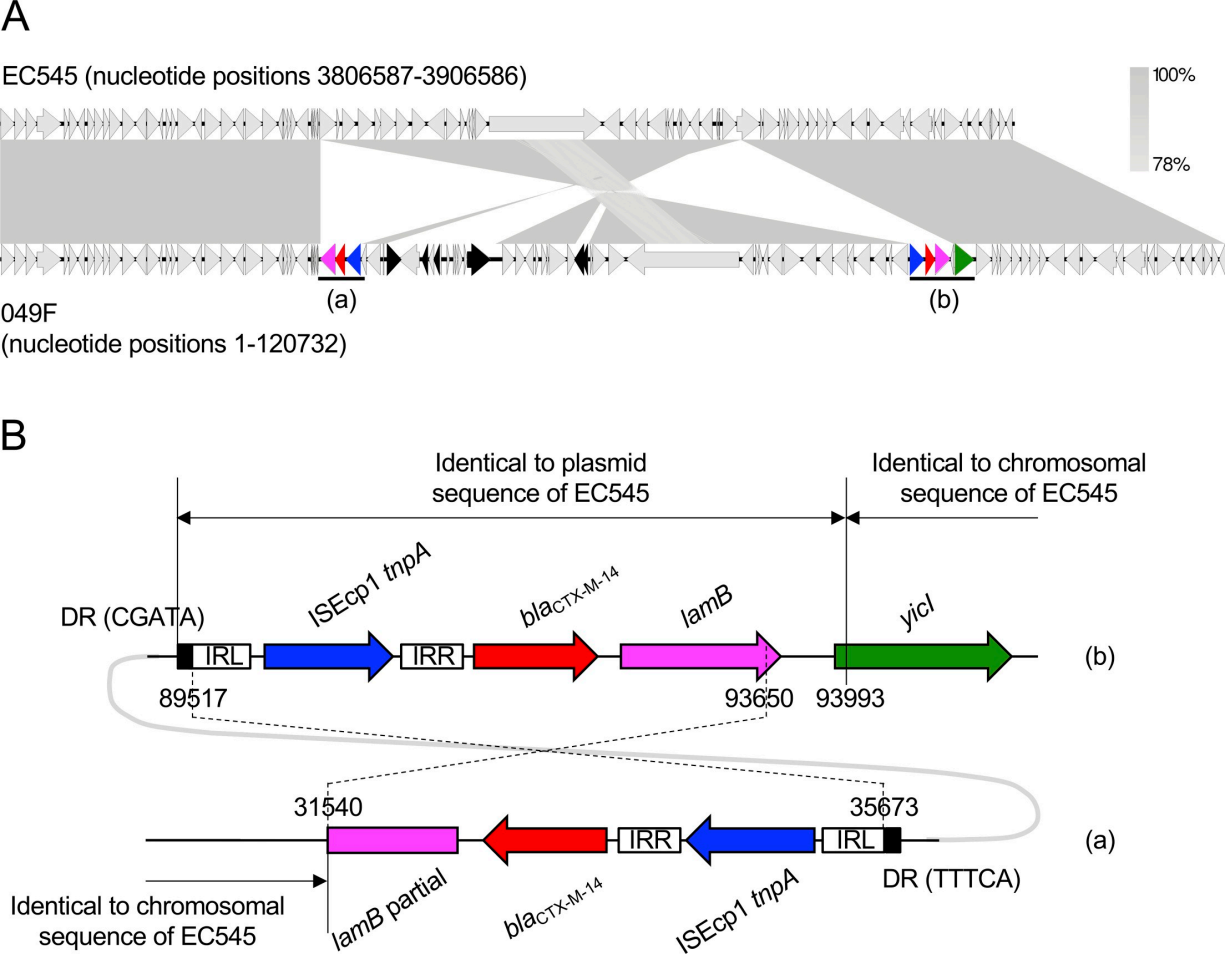
Characteristics of a chromosomal segment coding for *bla*_CTX-M-14_ from an *E*. *coli* isolate. The partial chromosomal sequence from strain 049F was compared with that from the most closely related *E*. *coli* strain 545 (accession no. NZ_CP018976). Homologous regions are shaded gray. Genetic elements are indicated by colors as follows: *bla*_CTX-M-14_, red; *lamB*, pink; *yicI*, green; ISEcp1 *tnpA*, blue; other mobile genetic elements, black. Regions (a) and (b) are enlarged in Fig 3B. (B) Details of the *bla*_CTX-M-14_ transposon elements of strain 049F in Fig 3A. Numbers indicate chromosomal nucleotide positions. DRs and IRs indicate direct repeats and inverted repeats, respectively. Nucleotide positions 89517–93650 are identical to positions 31540–35673. Nucleotide sequences of each IR are as follows: ISEcp1-IRL, CCTAGATTCTACGT; ISEcp1-IRR, CGTGGAATTTAGGG. The accession number for pEC545 is CP018973.

In several bacterial species, chromosomal *bla*_CTX-M_ (e.g., *bla*_CTX-M-2_, *bla*_CTX-M-14_, and *bla*_CTX-M-15_) were identified likely because of the transposition of *bla*_CTX-M_ from a plasmid into the chromosome [98–100]. The isolate 049F (ST38) possessed two chromosomal copies of *bla*_CTX-M-14_ (Fig 3A). The most closely related *E*. *coli* strain, 545, possessed the plasmid pEC545, in which the transposon element, ISEcp1-*bla*_CTX-M-14_-*lamB*, shared 100% homology with that in strain 049F (nucleotide positions 89517–93993 in Fig 3B). These results suggested that the *bla*_CTX-M-14_ gene in strain 049F was derived from a plasmid-borne transposon element, although the target site duplication (DR in Fig 3B) could not be identified. The other transposon element harboring *bla*_CTX-M-14_ in strain 049F (nucleotide positions 31540–35673 in Fig 3B) was identical to, but 343 bp shorter than, the larger element (nucleotide positions 89517–93993). These results suggested that the larger element was partially transferred (e.g., nucleotide positions 89517–93650 in Fig 3B) into the chromosome by the recurrent transposition that was observed in *E*. *coli* isolates with chromosomal *bla*_CTX-M-14_ [101]. The larger transposon element was inserted adjacent to *yicI*. Hamamoto *et al*. [101] found five genes adjacent to the insertion of a *bla*_CTX-M-14_ transposition element: *tetC*, *ompN*, *yhjQ*, *sraG*, and *dacD*. This study newly identified the *yicI* gene adjacent to the ISEcp1-*bla*_CTX-M-14_-*lamB* transposon element. Chromosomal *bla*_CTX-M-14_ was detected in various STs of human-derived *E*. *coli* isolates [98]. To the best of our knowledge, this was the first report of ST38 carrying chromosomal *bla*_CTX-M-14_, although ST38 possessing a *bla*_CTX-M-14_-coding plasmid was previously reported [64]; and therefore, the stability of chromosomal *bla*_CTX-M-14_, and its ability for cross-species transmission remain unclear. The chromosomal location of *bla*_CTX-M-14_ is considered to be one factor contributing to the high prevalence of these *E*. *coli* isolates in humans [98]. Continual surveillance in companion dogs is needed.

#### (iv) Plasmid harboring *bla*_CMY-2_

The most commonly reported AmpC β-lactamase is CMY-2. Plasmid-borne CMY-2 is distributed worldwide particularly in *E*. *coli* and *Salmonella* spp. from humans and animals [14,102]. Livestock animals, poultry in particular, are considered reservoirs for these bacteria and are a possible source of *bla*_CMY-2_ in humans [10]. This study showed that 42.9% of strains (6 of 14) possessed the plasmid harboring *bla*_CMY-2_ (Table 4). We did not determine whether the *bla*_CMY-2_ gene in the isolate 024S was located on the chromosome or a plasmid, because we failed to determine the whole chromosomal sequence. These results suggest that plasmid-borne CMY-2 is disseminated among companion dogs in Osaka, Japan. Various plasmid types, including IncA/C, IncI1, IncI2, and IncFII are associated with the presence of *bla*_CMY-2_ [90]. IncA/C and IncI1 plasmids, in particular, are often reported as the most common carriers of *bla*_CMY-2_ [90,103]. Of the six plasmids in this study, two (026F3c3 and 112Sc2) belonged to the IncI1 group. Two other plasmids (019Sp3 and 066Sp3) belonged to the IncFII group. The remaining two plasmids (128Sp3 and 128Fc7) belonged to the IncC (formerly IncA/C2) group.

**Fig 4:**
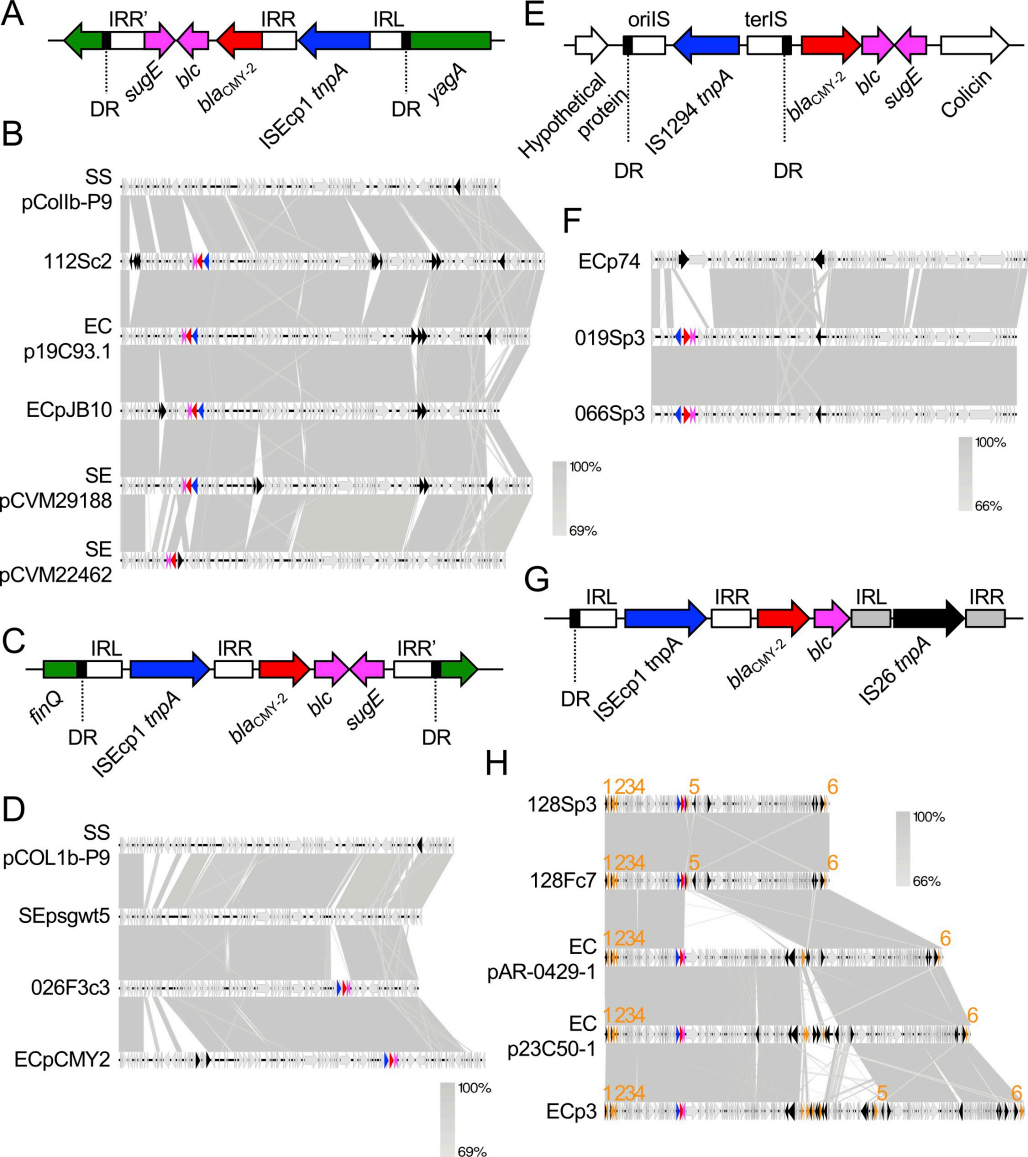
Characteristics of *bla*_CMY-2_-coding plasmids. (A) (C) (E) (G) Details of the *bla*_CMY-2_ transposon elements of the plasmids 112Sc2 (A), 026F3c3 (C), 019Sp3 and 066Sp3 (E), and 128Sp3 and 128Fc7 (G). DRs indicate 4–5-bp direct repeats: TGGGT (A), GATAA (C), GTTC (E), and ATTTC (G). IRs indicate inverted repeats. oriIS and terIS in Fig 4E are oriented and terminated insertion sequences, respectively. (B) (D) (F) (H) The plasmids encoding *bla*_CMY-2_ were compared with a prototype and highly homologous plasmids from *E*. *coli* (EC), *S*. *enterica* (SE), and *S*. *soneii* (SS). Genetic elements are indicated by colors as follows: *bla*_CMY-2_, red; other resistance genes, orange; *blc* and *sugE*, pink; genes disrupted by the ISEcp1-*bla*_CMY-2_ cassette, green; *tnpA* genes for ISEcp1 and IS1294, blue; other mobile genetic elements, black. Genes are indicated by numbers as follows: *tetA*, 1; *aph(6)-Id*, 2; *aph(3′′)-Ib*, 3; *sul1*, 4; *mph(A)*, 5; *floR*, 6. Nucleotide sequences of each IR are as follows: ISEcp1-IRL, CCTAGATTCTACGT; ISEcp1-IRR, ACGTGGAATTTAGG; ISEcp1-IRR’ in Fig 4A, GAAGCGTTTGCAGG; ISEcp1-IRR’ in Fig 4C, AACCAGAAAGTCGA; IS26-IRL, GGCACTGTTGCAAA; IS26-IRR, TTTGCAACAGTGCC; IS1294-oriIS, ACGTATAGGAAATTGAAAAAC; IS1294-terIS, TTGCAGCCGCCAGGCTGCCGTG. Accession numbers of database-derived plasmids are as follows: pColIb-P9, AB021078; p19C93.1, LC501481; pJB10, KX452392; pCVM29188, NC011077; pCVM22462, CP009566; psg_wt5, CP037994; pCMY2, LC019731; p74, CP023389; pAR-0429-1, CP044142; p23C50-1, LC501563; p3, CP031549.

We first focused on the IncI1 incompatibility group (plasmids 026F3c3 and 112Sc2). IncI1 has become one of the most common plasmid families in contemporary Enterobacteriaceae from both human and animal sources, and was confirmed as a transmitter of AmpC genes, particularly the *bla*_CMY-2_ gene, in isolates from livestock animals [104]. The 112Sc2 plasmid belonged to the pMLST12 which, in the IncI1 incompatibility group, has especially contributed to the wide spread of *bla*_CMY-2_ [104], and now features an epidemic plasmid in poultry and poultry products [10,105]. In the USA, the IncI1/pMLST12 plasmid harboring *bla*_CMY-2_ was detected in several companion dogs [106]. The *bla*_CMY-2_ gene of 112Sc2 was within a transposon-like structure consisting of ISEcp1-*bla*_CMY-2_-*blc*-*sugE* (Fig 4A), which is well conserved in IncI1/pMLST12 plasmids harboring *bla*_CMY-2_ in *E*. *coli* and *S*. *enterica* strains [107,108]. The transposon element was inserted in the *yagA* gene of the IncI1 prototype plasmid pColIb-P9 [109] from *S*. *sonnei*, flanked by the 5-bp DR (Fig 4A). The 112Sc2 plasmid exhibited 97.94% identity and 87% query coverage with pColIb-P9 (pMLST11), and was closely related to several pMLST12 plasmids: p19C93.1 from avian-derived *E*. *coli* in Japan in 2007 (99.91% identity and 94% query coverage), pJB10 [105] from avian-derived *E*. *coli* in Brazil in 2015 (99.99% identity and 88% query coverage), and pCVM29188 [110] from avian-derived *S*. *enterica* in the USA in 2003 (99.99% identity and 97% query coverage) (Fig 4B). The 112Sc2 plasmid was also highly similarity (99.81% identity and 84% query coverage) to the pMLST23 plasmid, pCVM22462 [106], from an *S*. *enterica* isolate from a dog. The other IncI1 plasmid, 026F3c3, was untypable, likely because the whole plasmid sequence was not determined. The transposon-like element ISEcp1-*bla*_CMY2_-*blc*-*sugE* was conserved, as in 112Sc2, but the element was inserted in the *finQ* gene, flanked by a 5-bp DR (Fig 4C). The disruption of the *finQ* gene by the *bla*_CMY-2_ transposon element was first reported in the *S*. *enterica* serotype Choleraesuis SCB67 strain in 2004 [111]. In addition, Su *et al*. [107] revealed that the transposon element carrying *bla*_CMY-2_ was inserted in the *finQ* gene of the transfer region of pColIb-P9 in most of the tested Enterobacteriaceae isolates, including *K*. *pneumoniae*, *E*. *coli*, *S*. *enterica*, *Citrobactor* spp., *S*. *sonnei*, and *M*. *morganii*. The 026F3c3 plasmid did not share high homology with the plasmid from *S*. Choleraesuis SCB67 (99.69% identity and 47% query coverage), suggesting that 026F3c3 was not directly related to the plasmid from *S*. Choleraesuis SCB67. The *finQ* gene appears to be a hot spot for insertion of the *bla*_CMY-2_ transposon element. Comparison of whole plasmid sequences revealed that 026F3c3 shared 99.23% identity and 88% query coverage with pColIb-P9 (Fig 4D). One of the plasmids most closely related to 026F3c3 was psg_wt5, from avian-derived *S*. *enterica* in Singapore in 2016 (99.53% identity and 93% query coverage), which lacked a *bla*_CMY-2_ transposon element. Plasmid 026F3c3 also shared high homology (98.67% identity and 96% query coverage) with pCMY-2 from human-derived pathogenic *E*. *coli* in Taiwan in 2013, which can be transformed into other bacterial species, such as *K*. *pneumoniae* [112]. We could not analyze 024S, because it was unclear whether *bla*_CMY-2_ was located on the chromosome or a plasmid, although the partial sequence was highly homologous to that in the 026F3p3 plasmid. For both 112Sc2 and 026F3c3, highly homologous plasmids were identified in unrelated bacterial species isolated in different countries and eras, suggesting that the plasmids have been spreading in several continents over many years, hosted by both zoonotic pathogens and commensal bacterial species, circulating in both humans and animals, including companion dogs.

We next focused on the IncFII/pMLST F40:A−:B− plasmids 019Sp3 and 066Sp3 from different companion dogs. The transposon element IS1294-*bla*_CMY-2_-*blc*-*sugE* was conserved in the two plasmids. Furthermore, this element was inserted between the genes encoding a hypothetical protein and colicin, flanked by a 4-bp DR in both plasmids [113] (Fig 4E). The two plasmids shared 100% homology; moreover, the core-genome SNP count was three between 019S and 066S (S2 Table). These results indicate that the ST162 *E*. *coli* isolates found carrying the IncFII/pMLST F40:A−:B− plasmid with *bla*_CMY-2_ can spread among companion dogs. These isolates may be prevalent (or outbreak) among companion dogs in Osaka, Japan. Blastn search showed that one of the most closely related plasmids, p74, from *E*. *coli* strain 1105 (97.96% identity and 86% query coverage) was derived from a dog that lacked the *bla*_CMY-2_ transposon element (Fig 4F). The most closely related plasmid from the human-derived *E*. *coli* strain 2014C-3741 shared less homology (95.10% identity and 81% query coverage) with that from the dog-derived strain. The plasmids from other bacterial species also showed low homologies: 93.92% identity and 74% coverage with pEG356 from *S*. *sonnei*, 96.63% identity and 66% coverage with pKP12226 from *K*. *pneumoniae*, 98.57% identity and 80% coverage with p12-4374_62 from *S*. *enterica*. Furthermore, to the best of our knowledge, ST162 *E*. *coli* is not prevalent among humans [14,114–116]. Thus, there is no evidence of ST162 carrying the IncFII/pMLST F40:A−:B− plasmid with *bla*_CMY-2_ being transferred between humans and companion dogs.

The other plasmids, 128Sp3 and 128Fc7, were derived from the same dog. They shared 100% sequence homology and belonged to the IncC (formerly IncA/C2) group. Genetic structures surrounding *bla*_CMY-2_ consisted of an ISEcp1 element upstream, and *blc* and IS26 downstream [117,118] (Fig 4G). Fig 4A, 4C and 4E and previous studies [14,107,108] strongly suggest that the *bla*_CMY-2_ transposon element is composed of IS-*bla*_CMY-2_-*blc*-*sugE*. The *sugE* gene, DR, and IRR’ for ISEcp1 in the 128Sp3 and 128Fc7 plasmids appeared to be disrupted by the insertion of the IS26-*mph(A)* element, although the duplicated target sequences for IS26 were not identified in this study. Disruption of the *sugE* gene by the IS26-*mph(A)* element indicated that *bla*_CMY-2_ was present before *mph(A)* was inserted. The two plasmids also harbored genes for resistance to tetracycline (*tetA*), aminoglycosides (*aph(6)-Id* and *aph(3′′)-Ib*), sulfonamide (*sul2*), macrolides (*mph(A)*), and chloramphenicol (*floR*) (Fig 4H). IncC plasmids are often reported as one of the most common carriers of *bla*_CMY-2_ [103,119]. We therefore examined the whole plasmid sequence of our plasmids for homology with those from the NCBI database. The plasmids pAR0429-1 from human-derived *E*. *coli* in the USA, p23C50-1 from avian-derived *E*. *coli* in Japan in 2011, and p3 from swine-derived *E*. *coli* cq9 in China in 2012 were highly homologous (nucleotide identity, 99.94%, 99.61%, and 99.98%, respectively; query coverage, 96%, 100%, and 98%, respectively) with 128Sp3 and 128Fp7 (Fig 4H). All the highly homologous plasmids possessed the intact transposon element ISEcp1-*bla*_CMY-2_-*blc*-*sugE*, and had conserved resistance genes *tetA*, *aph(6)-Id*, *aph(3′′)-Ib*, *sul1*, and *floR*. The most variable position was that contiguous to the *bla*_CMY-2_ transposon element. The plasmids pAR-0429-1, p23C50-1, and p3 had a cassette >56 kb that contained genes for conjugative transfer, ferredoxin, DNA methyltransferase, and drug resistance. The role of this cassette remains unclear, and it might restrict plasmid transfer between humans and animals. This type of plasmid possesses multidrug-resistance genes; therefore, continuous surveillance for cross-species transmission is needed.

#### (v) Virulence genes

Companion animals are thought to be reservoirs of human ExPEC, because closely related or indistinguishable human ExPEC strains were recovered from humans and their companion animals [71,73,120]. We therefore focused on virulence-associated genes in *E*. *coli* isolates for which the whole genome was analyzed (Table 5). Isolation of an *E*. *coli* strain from a patient with an extraintestinal infection does not, by itself, confer the designation of ExPEC, because commensal *E*. *coli* strains can participate in extraintestinal infections when an aggravating or unfavorable factor is present, such as a foreign body, a compromised host, or a high-density or mixed bacterial species inoculum [63,121]. Therefore, all isolates were screened for human ExPEC status using an established molecular definition consisting of the presence of more than two of five human ExPEC-defining markers, i.e., *papAH* and/or *papC*, *sfa* and/or *foc*, *afa* and/or *dra*, *iutA*, and *kpsMII* [122]. We could not determine whether the isolates were canine ExPECs, because virulence-associated genes have different prevalences in humans and animals [123], and there are no molecular definitions for canine ExPEC. The following eight isolates belonged to the human ExPEC group: four ST131 C1-M27 isolates carrying IncF/pMLST F1:A2:B20 with *bla*_CTX-M-27_ (026F2, 048F, 056S, and 082S), an ST68 isolate carrying IncI1 with *bla*_CMY-2_ (026F3), an ST405 isolate carrying IncFII_K_/pMLST K2:A−:B− with *bla*_CTX-M-15_ (123S), and two ST998 isolates carrying IncC/pMLST 3 with *bla*_CMY-2_ and IncI1/pMLST 16 with *bla*_CTX-M-15_ (128S and 128F). Human ExPEC ST131 isolates, in particular, showed extensive spread among human and companion animals [115], and ST131 C1-M27 isolates carrying *bla*_CTX-M-27_ in this study were closely related to those derived from humans (Fig 1 and S4 Table), strongly suggesting that certain *E*. *coli* isolates that produce ESBLs are transmitted between humans and companion dogs, and cause extraintestinal infections in both host species. The remaining five isolates were not human ExPECs: ST162 carrying *bla*_CMY-2_ (019S, 024S, and 066S), ST38 carrying *bla*_CTX-M-14_ (049F), and ST10 carrying *bla*_CMY-2_ (112S). We consider ST162 carrying the IncFII/pMLST F40:A−:B− plasmid with *bla*_CMY-2_ were likely not transmitted between humans and companion dogs as described above under ‘Plasmid harboring *bla*_CMY-2_’, because, to the best of our knowledge, there is no report of ST162 *E*. *coli* being prevalent in humans [14,114–116]. However, there is another possibility; ST162/*bla*_CMY-2_ can be transferred between humans and companion dogs, and causes extraintestinal infections in companion dogs but not in humans, because ST162 is not human ExPEC. It is essential to evaluate not only pathogenic but also commensal *E*. *coli* to elucidate the precise role of companion dogs in cross-species transmission.

In conclusion, whole-genome analysis revealed that companion dogs in Osaka, Japan possess *bla* genes that were diverse in genotype, location, and type of carrying plasmid. Certain *E*. *coli* isolates producing ESBL/AmpC (i.e., ST131/*bla*_CTX-M-27_) appeared capable of cross-species transmission and causing extraintestinal infections in both host species. By contrast, other isolates (e.g., ST162/*bla*_CMY-2_) were likely not transmitted between humans and companion dogs. These results suggest that specific *E*. *coli* strains can spread between humans and companion dogs. Previous reports on methicillin-resistant *Staphylococcus aureus* (MRSA) and vancomycin-resistant *Enterococcus* spp. (VRE) reflect this idea; a specific sequence type of MRSA was shared between companion animals and humans with no apparent adaptation to animals [124], and a particular form of a transposon described in only human VRE was found in VRE isolated from a dog [124]. High homologies between the tested dog-derived plasmids and those originating from other hosts, in particular, humans, and their horizontal transfer abilities (Table 6) strongly suggest that plasmids harboring the *bla* gene (i.e., IncFII/F1:A2:B20/*bla*_CTX-M-27_, IncI1/16/*bla*_CTX-M-15_, IncI1/*bla*_CMY-2_) can spread among humans, companion dogs, and other animals. Further investigation is needed to confirm cross-species transmission of bacteria or plasmids encoding ESBL or AmpC β-lactamase between humans and companion animals.

## Supporting information

S1 Fig Dendrogram of ESBL/AmpC-producing *E*. *coli* isolates from companion dogs. XbaI-digested genomes of 19 ESBL/AmpC-producing *E*. *coli* isolates from companion dogs were subjected to PFGE. The dendrogram shows genotypic relatedness. The scale bar represents percent similarity. S, extraintestinal specimen; F, fecal sample.
(PDF)

S1 Table Summary of whole-genome sequences of 14 *E*. *coli* isolates from companion dogs. (XLSX)

S2 Table Pairwise SNP matrix among whole-genome sequenced *E*. *coli* isolates from companion dogs. *E*. *coli* isolates 019S, 026F2, 026F3, and 128S were used as reference strains to call SNPs in each ST. Numbers in the distance matrix denote the numbers of SNPs between each sample pair.
(XLSX)

S3 Table Source tracking of whole-genome sequenced *E*. *coli* isolates from companion dogs. The closely-related isolates were screened using the genome-based SNP strategy of the single genome analysis tool on the BacWGST database.
(XLSX)

S4 Table Pairwise SNP matrix among ESBL-producing *E*. *coli* ST131 isolates. *E*. *coli* JJ1886 was used as a reference strain to call SNPs. Numbers in the distance matrix denote the numbers of SNPs between each sample pair.
(XLSX)
