# Supplementary figures and images for: Whole-genome analyses of extended-spectrum or AmpC β-lactamase-producing Escherichia coli isolates from companion dogs in Japan
Source: PLoS One. 2021 Feb 5;16(2):e0246482. doi: 10.1371/journal.pone.0246482 (PMC7864471; doi:10.1371/journal.pone.0246482)

S1 Figure

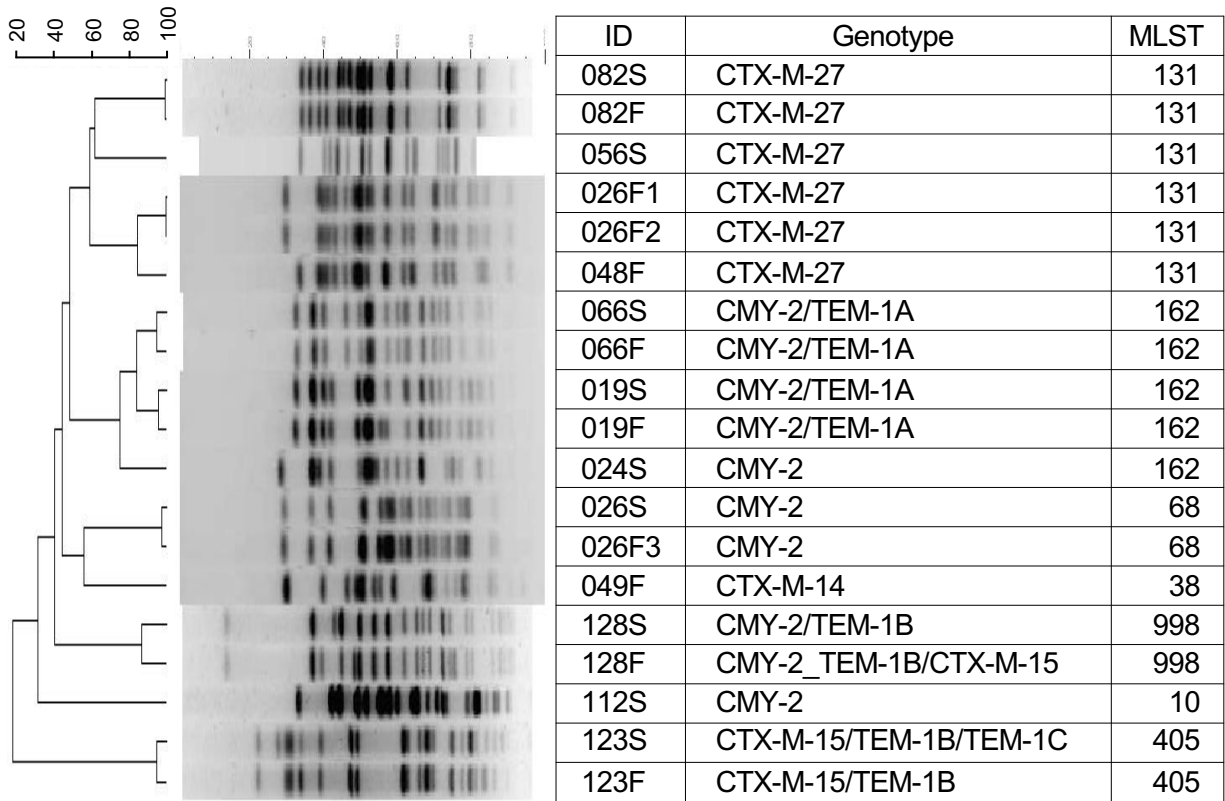

Supplement: S1 Fig — XbaI-digested genomes of 19 ESBL/AmpC-producing E. coli isolates from companion dogs were subjected to PFGE. The dendrogram shows genotypic relatedness. The scale bar represents percent similarity. S, extraintestinal specimen; F, fecal sample. (PDF) [file pone.0246482.s001.pdf]
